# Dispersion and stability mechanism of Pt nanoparticles on transition-metal oxides

**DOI:** 10.1038/s41598-022-17638-6

**Published:** 2022-08-11

**Authors:** Eun-Suk Jeong, In-Hui Hwang, Sang-Wook Han

**Affiliations:** 1 Department of Physics Education and Institute of Fusion Science, Jeonbuk National University, Jeonju, 54896 Korea; 2 X-Ray Science Division, Advanced Photon Source, Argonne National Laboratory, Lemont, IL 60439 USA

**Keywords:** Materials science, Nanoscience and technology

## Abstract

The heterogeneous catalysts of Pt/transition-metal oxides are typically synthesized through calcination at 500 °C, and Pt nanoparticles are uniformly and highly dispersed when hydrogen peroxide (H_2_O_2_) is applied before calcination. The influence of H_2_O_2_ on the dispersion and the stability of Pt nanoparticles on titania-incorporated fumed silica (Pt/Ti–FS) supports was examined using X-ray absorption fine structure (XAFS) measurements at the Pt L_3_ and Ti K edges as well as density functional theory (DFT) calculations. The local structural and chemical properties around Pt and Ti atoms of Pt/Ti–FS with and without H_2_O_2_ treatment were monitored using in-situ XAFS during heating from room temperature to 500 °C. XAFS revealed that the Pt nanoparticles of H_2_O_2_-Pt/Ti–FS are highly stable and that the Ti atoms of H_2_O_2_-Pt/Ti–FS support form into a distorted-anatase TiO_2_. DFT calculations showed that Pt atoms bond more stably to oxidized–TiO_2_ surfaces than they do to bare- and reduced–TiO_2_ surfaces. XAFS measurements and DFT calculations clarified that the presence of extra oxygen atoms due to the H_2_O_2_ treatment plays a critical role in the strong bonding of Pt atoms to TiO_2_ surfaces.

## Introduction

Noble-metal catalysts have been widely used for various applications, including fuel cells with oxygen reduction reactions^1,2^ and hydrogen evolution reactions^3,4^; NO_x_ reduction techniques such as selective catalytic reduction and lean NO_x_ trapping^5–7^; and photocatalysis^8^. For practical applications of noble metal catalysts, there have been many efforts to improve the dispersion and stability of noble-metal catalysts^9–13^. Many research groups have proposed different strategies through which to obtain highly-dispersed noble-metal catalysts with high catalysis efficiency and stability. For enhanced catalysis efficiency, researchers have attempted to control the morphology of nanoparticles alloyed with various metals, including Pd, Co, Ni, and Ti^14–16^, and they have designed graphene-based noble-metal nanostructures^17,18^. Heterogeneous catalysts with supports have also been extensively studied for high dispersion, because their structural and chemical properties can be modified for optimum dispersion^19–23^. For practical applications to heterogeneous catalysts, various transition-metal-oxide supports, including TiO_x_, CeO_x_, and ZrO_x_, have been examined to enhance catalysis efficiency and selectivity^24–28^. Agglomeration of noble-metal particles on transition-metal-oxide supports is frequently observed during synthesis due to the use of a high-temperature sintering process^9–11^. Kim and co-workers showed uniformly-dispersed Pt nanoparticles with a mean diameter of less than 1 nm on transition-metal-oxide supports^9,10,29^. They argued that the agglomeration of noble-metal particles on transition-metal-oxide supports could be avoided using a hydrogen peroxide (H_2_O_2_) treatment. However, the dispersion and stability mechanism of noble metal on transition-metal-oxide supports with an H_2_O_2_ treatment during the synthesis process remains unclear and needs to be elucidated.

H_2_O_2_ treatments have been widely used in various fields, including organic and inorganic chemical sciences, material science, and biological science. Previous studies have shown that H_2_O_2_ treatments could alter the oxidation states and local structural properties around transition-metal atoms^30–39^. It could be due to the strong oxidizing characteristics of H_2_O_2_^31–33,37^. Researchers have reported that H_2_O_2_ treatments cause a red shift of the ZnO bandgap and enhance the crystal quality of ZnO nanorods^34,35^. With H_2_O_2_ treatments, the improved morphology of ZrO_2_ particles^40^, the crystal structure and the exposed surface changes of TiO_2_^34^, and the aggregation of amorphous calcium phosphate nanoparticles^37^ were observed as well. Previous studies strongly suggested that H_2_O_2_ treatments significantly affect both the outermost surfaces and internal structures of materials without matter of the crystallization. Microscopic measurements are needed to understand the influence of H_2_O_2_ on the surfaces, interfaces, and internal structures of the materials. Heterogeneous Pt/TiO_2_ catalysts are widely used for practical applications. Pt nanoparticles are highly dispersive and quite stable on TiO_2_ supports when H_2_O_2_ treatment is used. In this study, we examined the effects of H_2_O_2_ on the bonds of Pt nanoparticles to TiO_2_ supports to understand the dispersion and stability of noble-metal catalysts on transition-metal-oxide supports.

The influence of H_2_O_2_ on the stoichiometric change, morphology, and crystal structure of metal nanoparticles has been examined using scanning electron microscopy (SEM), transmission electron microscopy (TEM), and X-ray diffraction (XRD) measurements^30,34,37^. However, SEM, TEM, and XRD measurements are limited in their quantitative characterizations of the effects of H_2_O_2_ treatment on the dispersion of and local structural change in metal nanoparticles during the synthesis process, because most nanoparticles are amorphous and in the sub-nanometer scale in size. X-ray absorption fine structure (XAFS) is a suitable tool for investigating the local structural and chemical properties around a selected species atom of compounds. XAFS is particularly useful for in-situ examinations of the local structural and chemical property changes of heterogeneous compounds on the nanometer scale^11,21,41^. The local structural and chemical properties around the noble-metal and transition-metal atoms of noble metal/transition-metal-oxide catalysts were examined using in-situ XAFS measurements at the absorption edges of the noble-metal and the transition-metal atoms. As a complement to the XAFS measurements, density functional theory (DFT) calculations were performed to elucidate the stability of the bonds between noble metal and metal-oxide support. We observed that the presence of extra oxygen atoms due to the H_2_O_2_ treatment plays an important role in the high dispersion and stability of Pt nanoparticles on TiO_2_ supports.

## Results

### Temperature-dependent XANES spectra

**Figure 1:**
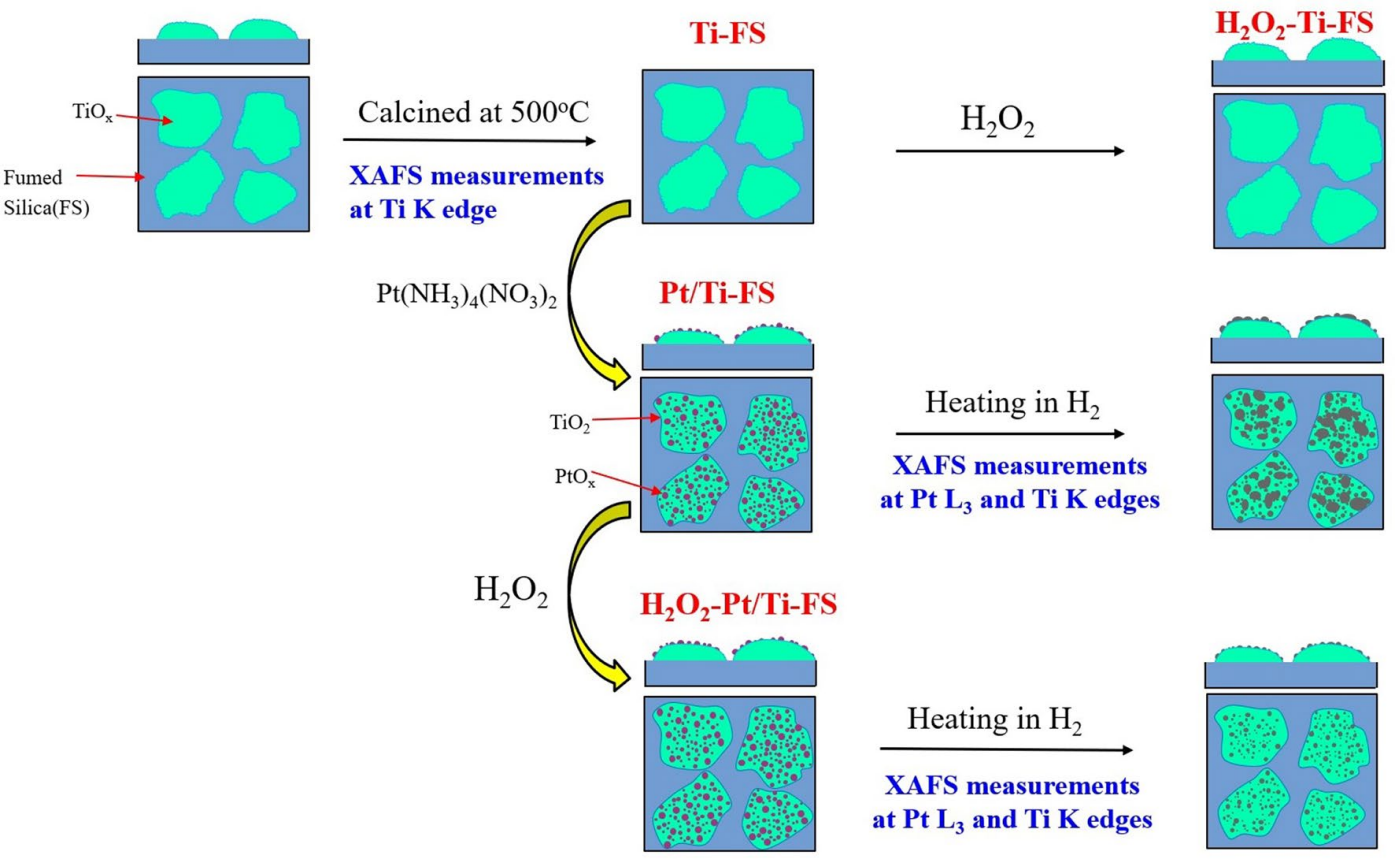
Synthesis procedure of Pt/Ti–FS, specimen names, and conditions of XAFS measurements.

**Figure 2:**
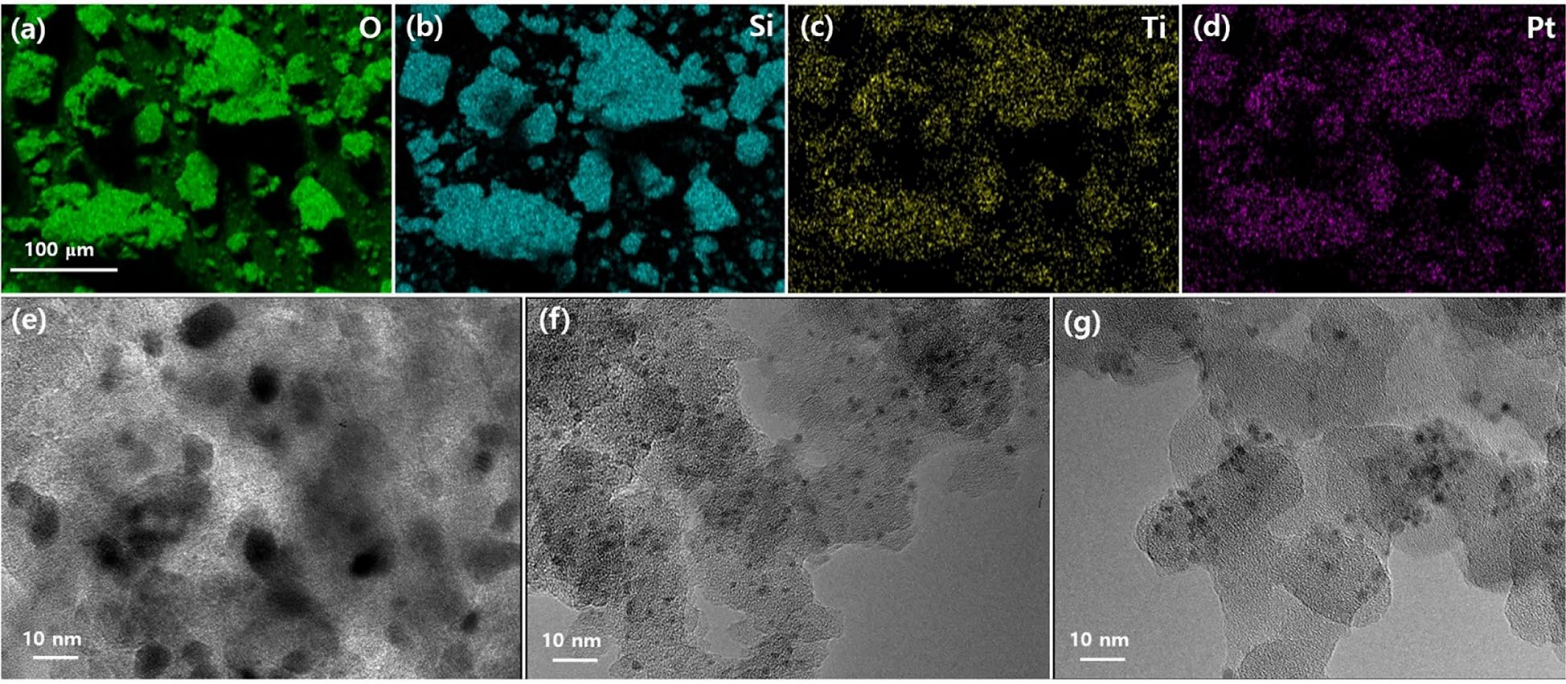
EDS images of H_2_O_2_-Pt/Ti–FS for (**a**) O, (**b**) Si, (**c**) Ti, and (**d**) Pt atoms. TEM images of (**e**) Pt/FS, (**f**) Pt/Ti–FS, and (**g**) H_2_O_2_-Pt/Ti–FS. The specimens were yielded after finally calcined at 500  °C under an H_2_ environment.

**Figure 3:**
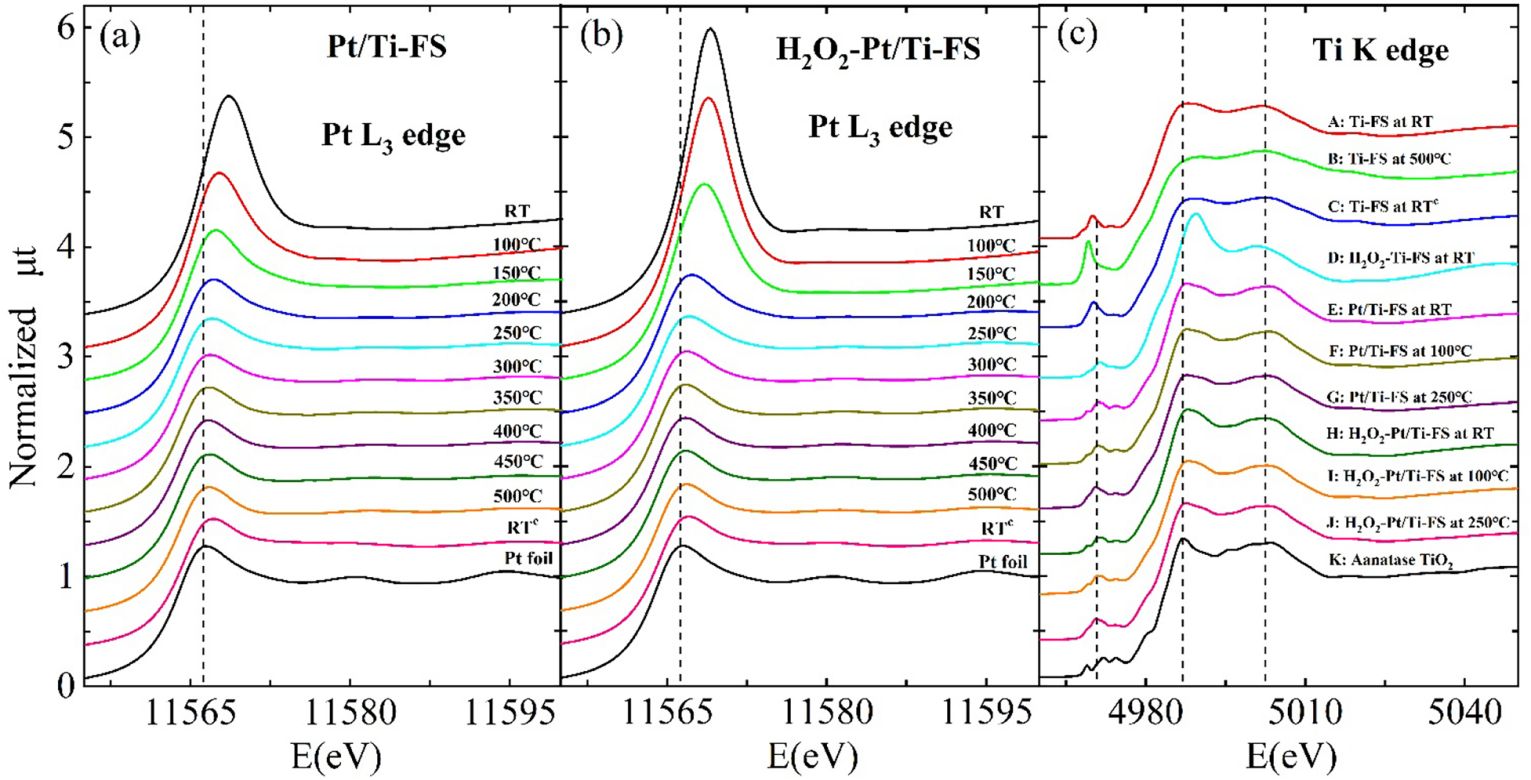
Normalized XANES (μt) spectra of (**a**) Pt/Ti–FS and (**b**) H_2_O_2_-Pt/Ti–FS at the Pt L_3_ edge at different temperatures. (**c**) XANES spectra at the Ti K edge of A: Ti–FS at RT, B: Ti–FS at 500 °C, C: Ti–FS at RT^c^, D: H_2_O_2_–Ti–FS at RT, E: Pt/Ti–FS at RT, F: Pt/Ti–FS at 100 °C, G: Pt/Ti–FS at 250 °C, H: H_2_O_2_-Pt/Ti–FS at RT, I: H_2_O_2_-Pt/Ti–FS at 100 °C, J: H_2_O_2_-Pt/Ti–FS at 250 °C, and K: anatase TiO_2_ at RT. RT^c^ indicates RT after being heated up to 500 °C and cooled down. XANES measurements were taken under an H_2_ environment except for H_2_O_2_–Ti–FS and anatase TiO_2_, which were taken in air. The vertical dished lines are a guide to the eye.

For this study, Pt nanoparticles were synthesized on titania-incorporated fumed silica (Ti–FS) supports with and without H_2_O_2_ treatment^9,10^, as summarized in Fig. 1. The size and the distribution of Pt nanoparticles were examined by energy dispersive spectroscopy (EDS) and TEM measurements, as shown in Fig. 2. X-ray absorption near edge structure (XANES) is sensitive to the chemical valence state as well as the geometry of the nearest neighboring atoms around a probing atom^42,43^. Temperature-dependent XANES at the Pt L_3_ edge shows a dramatic change in the white line intensity during heating, as shown in Fig. 3a and b. Changes in the Pt white line are known to be directly related to Pt oxidation^11,44,45^. Jeong et al. clarified that the white line area of Pt L_3_ edge directly corresponds to the coordination number of oxygen atoms bonding to Pt atoms^11^. When a Pt atom bonds with oxygen atoms, the empty state density of the Pt 5d orbitals increases because the electrons in the Pt 5d orbitals transfer to the oxygen atoms. The strong intensity of the white line and the slight shift toward a higher energy of the Pt absorption edge of both Pt/Ti–FS and H_2_O_2_-Pt/Ti–FS at the room temperature (RT) indicate a high oxidation of Pt atoms compared to those of a Pt foil, as shown in Fig. 3a and b. The white line features strongly suggest that the Pt atoms on Ti–FS supports with no matter of the H_2_O_2_ treatment at RT are oxidized to PtO_x_. At RT, the intensity of the Pt white line of H_2_O_2_-Pt/Ti–FS is substantially stronger than that of Pt/Ti–FS, as shown in Fig. 3a and b, respectively. This indicates that the mean oxidation state of Pt atoms of H_2_O_2_-Pt/Ti–FS is higher than that of Pt atoms of Pt/Ti–FS, which is attributed to the H_2_O_2_ treatment. This agrees with the previous reports which showed an oxidizing agent of H_2_O_2_ to metals^31–33,37^. The intensity increase of the white line might suggest the change of the Pt precursor from [Pt(NH_3_)_4_](NO_3_)_2_ of the Pt/Ti–FS to [Pt(NH_3_)_4_(OH)_2_](NO_3_)_2_ of the H_2_O_2_-Pt/Ti–FS, although XAFS cannot determine the chemical formulas of the Pt precursors due to the resolution limit. When heated up to 250 °C, the intensities of the white lines of both Pt/Ti–FS and H_2_O_2_-Pt/Ti–FS decrease dramatically, and the absorption edges shift toward a lower energy, which is the nearly same as the absorption edge of a Pt foil at RT. The temperature-dependent behavior of the white line strongly implies that the Pt atoms of both Pt/Ti–FS and H_2_O_2_-Pt/Ti–FS are rapidly reduced in an H_2_ environment during heating, and that most of the oxygen atoms dissociate from the Pt nanoparticles at 250  °C.

The chemical valence state and the local structural properties around the Ti atoms of Pt/Ti–FS and H_2_O_2_-Pt/Ti–FS supports can be also changed with those around the Pt atoms during the synthesis and heating processes. A change around the Ti atoms of Ti–FS supports is crucial for understanding the dispersion mechanism of Pt nanoparticles on the supports. The changes of the chemical and structural properties around the Ti atoms of the Ti–FS supports can affect the dispersion and the stability of Pt nanoparticles on the supports. Figure 3c A–J show XANES spectra of Ti–FS specimens with different conditions at the Ti K edge. The main edge energy of the entire specimens is approximately 4982 eV, which is similar to that of anatase TiO_2_, as shown in Fig. 3c K. This implies that most of the Ti atoms of the specimens have the chemical valence state of 4^+^. Some differences of the pre-edge peaks as well as the main edges may indicate the slight variation of structural properties around the Ti atoms of the specimens. Figure 3c A–C show XANES spectra of Ti–FS supports during the temperature change of RT → 500  °C → RT^c^ in an H_2_ environment. The XANES spectra of the Ti–FS supports at RT and RT^c^ are somewhat different from each other, implying a slight change of the local structural properties around the Ti atoms of the Ti–FS supports for the calcining process. The XANES of H_2_O_2_–Ti–FS reveals that H_2_O_2_ treatment significantly affects the local structural properties around the Ti atoms of Ti–FS, compared to that of Ti–FS at RT^c^, as shown in Fig. 3c D and C, respectively. The increased intensity of the white line in Fig. 3c D indicates that an H_2_O_2_ treatment causes to increase the coordination of oxygen atoms around the Ti atoms of the supports. It is noted that the Ti–FS specimen was not calcined before the XAFS measurements, while the H_2_O_2_–Ti–FS specimen was yielded as applying H_2_O_2_ on calcined Ti–FS, as illustrated in Fig. 1. To elucidate the dispersion mechanism of Pt nanoparticles on the Ti–FS supports which have been calcined at 500  °C before the Pt precursor is impregnated, it is important to have a direct comparison of the XANES spectra of Pt/Ti–FS with and without H_2_O_2_ treatment at the Ti K edge as well as at the Pt L_3_ edge. Figure 3c E–G and H–J, respectively, show the XANES spectra of Pt/Ti–FS without and with H_2_O_2_ treatment at the Ti K edge. At RT, the XANES spectra of Pt/Ti–FS and H_2_O_2_-Pt/Ti–FS at the Ti K edge are similar to each other, while they are different from that of H_2_O_2_–Ti–FS, as shown in Fig. 3c D, E, and H. This finding suggests that the local structure around the Ti atoms of the Pt/Ti–FS does not significantly change due to the H_2_O_2_ treatment. The XANES spectra of Ti–FS at RT^c^, H_2_O_2_–Ti–FS, Pt/Ti–FS, and H_2_O_2_-Pt/Ti–FS at the Ti K edge indicate that the Ti atoms of calcined Ti–FS have a stable local structure, when the Pt precursor is impregnated. The XANES spectra suggest that the local structure around the Ti atoms of Pt/Ti–FS and H_2_O_2_-Pt/Ti–FS is close to an anatase TiO_2_. The structural properties around the probing Pt and Ti atoms of Pt/Ti–FS systems can be more clearly seen in more detail in the extended XAFS (EXAFS) at the Pt L_3_ and Ti K edges, respectively, which is a small oscillations above the absorption edges.

### Temperature-dependent local structural properties

**Figure 4:**
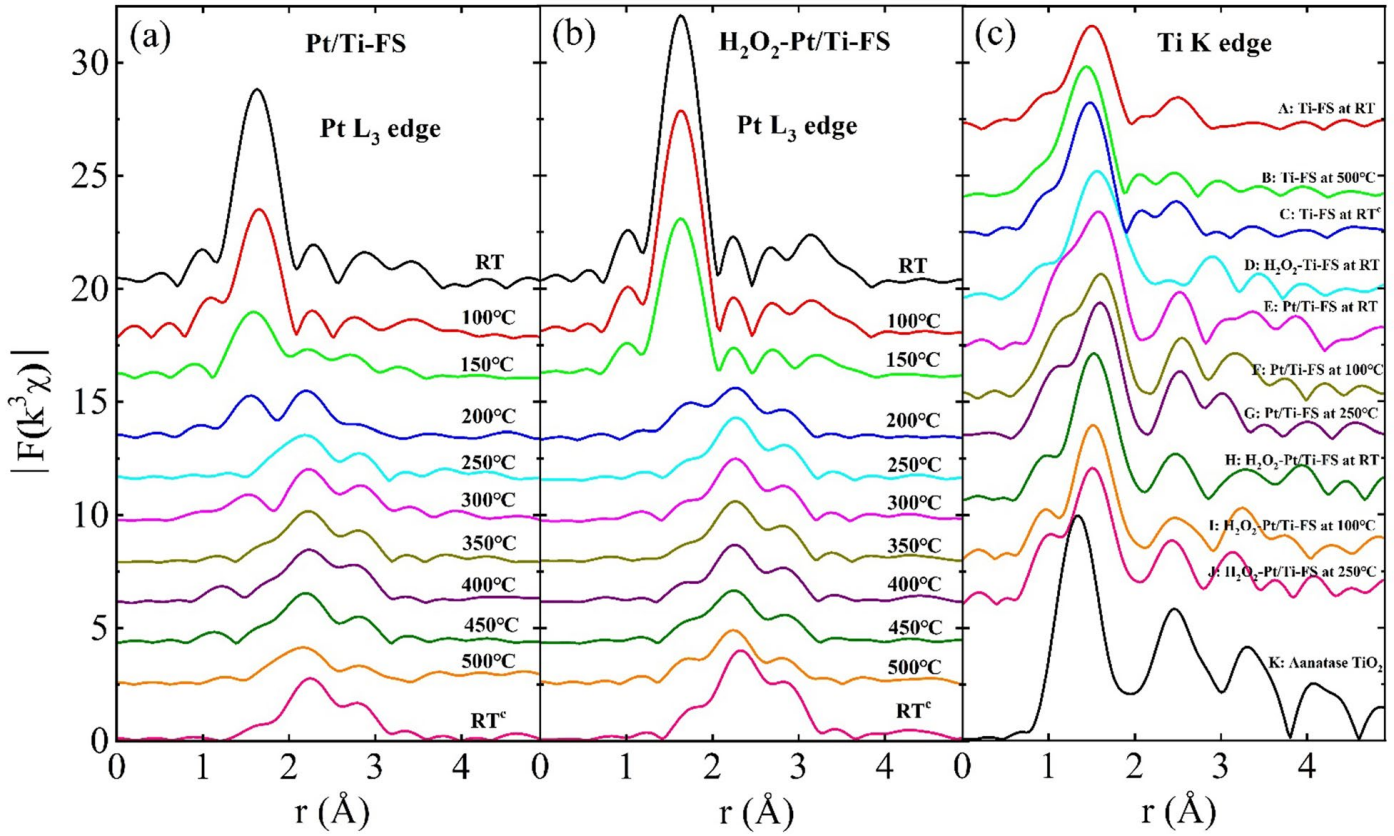
Magnitude of FT-EXAFS (|k^3^χ|) as functions of the distance from a Pt atom of (**a**) Pt/Ti–FS and (**b**) H_2_O_2_-Pt/Ti–FS at different temperatures. (**c**) EXAFSs as functions of the distance from a Ti atom of A: Ti–FS at RT, B: Ti–FS at 500 °C, C: Ti–FS at RT^c^, D: H_2_O_2_–Ti–FS at RT, E: Pt/Ti–FS at RT, F: Pt/Ti–FS at 100 °C, G: Pt/Ti–FS at 250 °C, H: H_2_O_2_-Pt/Ti–FS at RT, I: H_2_O_2_-Pt/Ti–FS at 100 °C, J: H_2_O_2_-Pt/Ti–FS at 250 °C, and K anatase TiO_2_ at RT. For the Fourier transform, the EXAFS data in the k-range from 2.5 to 10.5 Å^−1^ and a Hanning window with a windowsill with of 0.5 Å^−1^ were used.

EXAFS can be used to quantitatively determine the local structural properties around a selected species atom of compounds^46–48^. After atomic background function was determined using the AUTOBK code^49^, EXAFS data was extracted from XAFS and Fourier transformed to the r-space, as shown in Fig. 4. EXAFS was quantitatively analyzed with the IFEFFIT package^50^ using standard analysis procedures^48,51^. The peak positions of EXAFS data correspond to the atomic shell distances from a probing atom. The peak positions of the EXAFS data are approximately 0.3 Å shorter than the true distances of atomic pairs because the phase shift of back-scattered photoelectrons by neighboring atoms has not yet to be considered. At the Pt L_3_ edge, the temperature-dependent EXAFS of Pt/Ti–FS with and without H_2_O_2_ treatment reveals that the local structure around Pt atoms is significantly changed by heating under a H_2_ environment, as shown in Fig. 4a and b. At RT the first and second peaks of ~ 1.7 Å and 2.2 Å respectively correspond to O and Pt atoms^11^. The first peak intensity gradually weakens during heating, and it virtually disappears at 250  °C. This indicates that Pt atoms initially bond with oxygen atoms as well as Pt atoms, and that there is a lack of Pt-O bonds at high temperatures. After being heated up to 500  °C and cooled down to RT (RT^c^), the local structures around the Pt atoms of both Pt/Ti–FS and H_2_O_2_ Pt/Ti–FS at RT^c^ are nearly identical to those above 250  °C, strongly implying that Pt nanoparticles have a stable structure due to the calcining process. This result is consistent with the XANES measurements of Pt/Ti–FS and H_2_O_2_ Pt/Ti–FS. EXAFSs at the Pt L_3_ edge of both Pt/Ti–FS and H_2_O_2_ Pt/Ti–FS show prominent peaks at approximately 2.2 Å and 2.8 Å when heated above 200  °C; these peaks are expected to be Pt-Pt pairs. Above 200  °C, the lack of any change in the temperature-dependent EXAFS of Pt-Pt pairs strongly implies a stable structure of Pt atoms in particular in H_2_O_2_-Pt/Ti–FS. EXAFS measurements of H_2_O_2_-Pt/Ti–FS at the Pt L_3_ edge reveal that oxygen atoms wrapping the tops of Pt nanoparticles are mostly dissociated, while Pt atoms form into stable Pt nanoparticles, tightly bonded to the Ti–FS supports, when heated up to 250 °C.

EXAFS of Ti–FS at the Ti K-edge shows the first and second peaks at 1.7 Å and 2.5 Å, respectively, as shown in Fig. 4c A and C. These peaks respectively correspond to the Ti–O and Ti–Ti pairs of a TiO_2_ structure^52,53^. For the Ti–FS at RT, the intensity of the first peak at ~ 1.7 Å increases and the shape of the second peak at ~ 2.5 Å changes, compared with those at RT^c^. This corresponds to the XANES spectra, as shown in Fig. 3c A and C. The distance of the Ti–Ti pairs is expanded due to the H_2_O_2_ treatment, compared to that of Ti–FS at RT^c^, as shown in Fig. 4c C and D. EXAFS at the Ti K edge indicates that the TiO_x_ complexes of the Ti–FS at RT^c^ have a quite unstable structure, because the peak positions and shapes of EXAFS substantially depend on the H_2_O_2_ treatment. The contribution of the Pt precursor on the local structure around Ti atoms of the Ti–FS supports can be seen with the direct comparison of the Ti–FS at RT^c^ and the Pt/Ti–FS at RT. It is noted that the Ti–FS of the Pt/Ti–FS was calcined at 500  °C before the Pt precursor is impregnated. Figure 4c C and E show the EXAFS data of the Ti–FS at RT^c^ and the Pt/Ti–FS at RT, respectively. The first and second peaks of the Ti–O and Ti–Ti pairs of the Ti–FS at RT^c^ are substantially different from those of the Pt/Ti–FS at RT. This indicates that the Pt precursor considerably affects the local structure around Ti atoms of the calcined Ti–FS supports. The position of the second peak of the Pt/Ti–FS is similar to that of the second peak of the anatase TiO_2_. This suggests that the Ti–Ti pairs of the Pt/Ti–FS have a similar distance of those of the anatase TiO_2_. EXAFS of H_2_O_2_-Pt/Ti–FS shown in Fig. 4c H–J clearly shows three peaks at ~ 1.5 Å, ~ 2.3 Å, and ~ 3.2 Å, which are similar to those of the anatase TiO_2_. The third peak at ~ 3.2 Å of Pt/Ti–FS is substantially different from that of H_2_O_2_-Pt/Ti–FS, particularly at 250  °C, as shown in Fig. 4c G and J. The EXAFS peak positions of H_2_O_2_-Pt/Ti–FS at the Ti K edge show a lack of changes, whereas they exhibit some changes in Pt/Ti–FS during heating from RT to 250  °C. This finding suggests that an unstable-distorted TiO_2_ structure of Pt/Ti–FS becomes a stable-distorted TiO_2_ due to the H_2_O_2_ treatment. The quantitative local structural properties can be obtained by fitting the EXAFS data to the EXAFS theoretical calculations using a structural model^48,51^.

### Quantitative analysis of local structural properties

Using the standard fitting procedures, EXAFS data in the r-space were fitted to EXAFS theoretical calculations with different structural models at the Pt L_3_ and Ti K edges^48,51^. The structural models of EXAFS theoretical calculations were designed based on the measured XANES and EXAFS data. PtO_x_ and Pt foil structures were initially modeled for the low and high temperatures of both Pt/Ti–FS and H_2_O_2_-Pt/Ti–FS, respectively. Pt precursors likely have the chemical formulas of [Pt(NH_3_)_4_](NO_3_)_2_ and [Pt(NH_3_)_4_(OH)_2_](NO_3_)_2_ for the Pt/Ti–FS and H_2_O_2_-Pt/Ti–FS, respectively, at RT. EXAFS cannot distinguish between O and N atoms and cannot detect H atoms due to the resolution limit. Furthermore, the local structure around Pt atoms are substantially changed when heated, as shown in Fig. 4. Thus, we chose a PtO_x_ structural model for the EXAFS data fits at low temperatures. At intermediate temperatures, a mixture structure of PtO_x_ and Pt foil was used to fit EXAFS data. For the EXAFS data fitting of the Ti K edge, structural models were selected based on the XANES and EXAFS data of Ti–FS and Pt/Ti–FS. In the fitting of the EXAFS data of H_2_O_2_-Pt/Ti–FS, a distorted-anatase TiO_2_ structure was used for the EXAFS theoretical calculations. EXAFS theoretical calculations were done using the FEFF8 code^54^, and EXAFS data was fitted using the IFEFFIT package^50^. In the fittings, the distance, the coordination number, and the Debye–Waller factors (${\upsigma }^{2}$, including thermal vibration and static disorder) of each atomic shell were varied. Only single-scattered paths were included in the fittings; this decision was made because, due to the particle size and the structural disorder, the EXAFS signal of a multiple-scattered path of nanoparticles is much weaker than that of a single-scattered path. A k-weight fit was used to reduce the correlation between ${\upsigma }^{2}$ and coordination number^53^.

**Figure 5:**
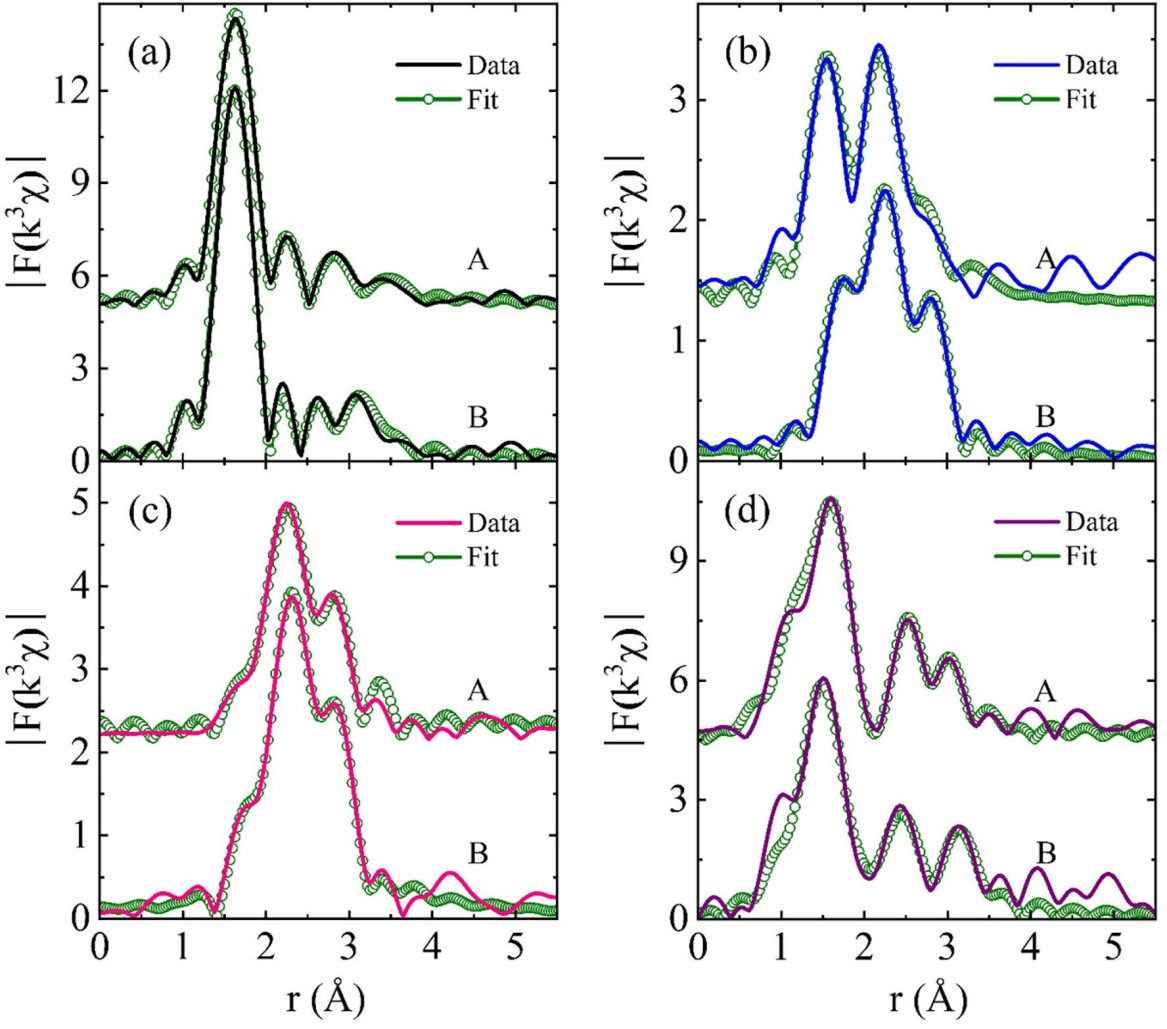
Representative FT-EXAFS data and the best fits. At the Pt L_3_ edge of A Pt/Ti–FS and B H_2_O_2_-Pt/Ti–FS at different temperatures of (**a**) RT, (**b**) 200  °C, and (**c**) RT^c^, respectively. (**d**) At the Ti K edge of Pt/Ti–FS and H_2_O_2_-Pt/Ti–FS at 250  °C. Data in the r-ranges from 1.3 – 3.5 Å and from 1.3 – 4.3 Å were respectively fitted for the Pt L_3_ and Ti K edges.

Figure 5 shows representative EXAFS data and the best fits. The quantitative structural properties around the Pt and Ti atoms of Pt/Ti–FS and H_2_O_2_-Pt/Ti–FS were obtained from the goodness fits of the EXAFS data. The results of the best fits are summarized in Tables 1–3. EXAFS at the Pt L_3_ edge reveals that a Pt atom of Pt/Ti–FS and H_2_O_2_-Pt/Ti–FS initially bonds with approximately five and six oxygen atoms, respectively. Pt atoms of Pt/Ti–FS initially have the second and third neighbors of ten Pt atoms at the respective distances of ~ 2.8 Å and ~ 3.1 Å. When heated above 250  °C, the Pt atoms of Pt/Ti–FS form into crystal nanoparticles with a substantial amount of structural disorder in the distance of Pt-Pt pairs, compared to that of the Pt foil. When H_2_O_2_ treatment is applied on Pt/Ti–FS at RT, Pt atoms have the first and second neighbors of six O and eight Pt atoms at the respective distances of ~ 2.0 Å and ~ 3.28 Å. In H_2_O_2_-Pt/Ti–FS, the coordination numbers of oxygen and Pt atoms around Pt atoms are increased and decreased, respectively, and the distance of Pt-Pt pairs is elongated, compared to those of Pt/Ti–FS. The bond lengths of Pt-Pt pairs of Pt metal and Pt/Ti–FS are 2.776 Å and 2.856 Å, respectively, while that is 3.28 Å in H_2_O_2_-Pt/Ti–FS, as shown in Tables 1 and 2. More oxidization of Pt atoms in H_2_O_2_-Pt/Ti–FS than in Pt/Ti–FS agrees well with the XANES measurements, as shown in Fig. 3a and b. This finding may suggest that the Pt precursor, [Pt(NH_3_)_4_](NO_3_)_2_, on the Ti–FS supports could form into [Pt(NH_3_)_4_(OH)_2_](NO_3_)_2_, when H_2_O_2_ is applied. As a result, the Pt atoms of Pt/Ti–FS at RT have more oxygen neighbors and the distance of Pt-Pt pairs is elongated. This corresponds to the previous studies of H_2_O_2_ treatment on transition-metal systems^31–33,37–39^. Pt nanoparticles can be more dispersed from [Pt(NH_3_)_4_(OH)_2_](NO_3_)_2_/Ti–FS than from [Pt(NH_3_)_4_](NO_3_)_2_/Ti–FS due to extra oxygen atoms, when they are calcined at 500  °C. This corresponds to the previous study of which Pt nanoparticles on Al_2_O_3_ supports were more dispersed when more oxygen atoms remained in the Pt precursor of [Pt(NH_3_)_4_](OH)_2_^55^. The role of oxygen atoms to separate metal atoms in atomic scale was also observed in Pd nanoparticles^56^. Our study suggests that the oxygen atoms around Pt atoms at the initial stage of the synthesis process play a decisive role in a high dispersion of Pt nanoparticles; meanwhile, they at the interface of Pt nanoparticles and Ti–FS supports assist a strong bond between Pt atoms and TiO_2_ supports when heated to a high temperature. When heated above 250  °C, the coordination number of Pt atoms of Pt/Ti–FS is gradually increased to be ~ 10 at 500  °C, whereas that of H_2_O_2_-Pt/Ti–FS shows a lack of changes in the temperature range of 250–500  °C. This result strongly implies that, at high temperatures, the Pt atoms of Pt/Ti–FS and H_2_O_2_-Pt/Ti–FS move to become lumpy and are pinned on the supports, respectively. A large σ^2^ value of the Pt-Pt pairs of Pt/Ti–FS indicates a less stable structure of Pt nanoparticles than that of H_2_O_2_-Pt/Ti–FS, particularly at RT^c^. When cooled down to RT from 500  °C, the local structural properties around Pt atoms of both Pt/Ti–FS and H_2_O_2_-Pt/Ti–FS are nearly the same as those at 500  °C, except for the σ^2^ values of Pt-Pt pairs due to the thermal effect. Jeong and co-workers demonstrated using EXAFS measurements that the Pt nanoparticles of H_2_O_2_-Pt/Ti–FS likely have a pancake shape on TiO_2_ supports^11^. In this case, Pt nanoparticles stably bond to the supports with a high catalysis efficiency^9^. At RT^c^, the bond lengths of the Pt-Pt pairs of both Pt/Ti–FS and H_2_O_2_-Pt/Ti–FS are shorter than that of a Pt foil due to the effects of nanoparticle boundaries with dangling bonds^57^.

**Table 1:** Fit results of EXAFS data of Pt/Ti–FS at the Pt L_3_ edge.

T ( °C)	Pt–O			Pt-Pt(1)				Pt-Pt(2)		
	*N*	*d* (Å)	σ^2^ (Å^2^)	*N*		*d* (Å)	σ^2^ (Å^2^)	*N*	*d* (Å)	σ^2^ (Å^2^)
RT (Pt-foil)				12.0(7)		2.776(3)	0.005(1)			
RT	5.0(6)	2.021(6)	0.003(1)	5(1)		2.856(9)	0.009(1)	5(1)	3.10(1)	0.010(1)
100	3.6(6)	2.036(7)	0.005(1)	4(1)		2.84(1)	0.013(1)	4(1)	3.08(1)	0.013(1)
150	1.8(6)	2.001(9)	0.005(1)	6(1)		2.73(1)	0.016(1)	2(1)	3.07(2)	0.022(5)
200	1.0(6)	1.94(1)	0.003(2)	8.4(8)		2.682(9)	0.016(1)			
250				8.6(8)		2.686(9)	0.015(1)			
300				8.4(8)		2.673(9)	0.014(1)			
350				8.4(8)		2.680(6)	0.014(1)			
400				8.2(9)		2.685(6)	0.014(1)			
450				9.0(9)		2.661(8)	0.015(1)			
500				9.6(9)		2.673(9)	0.018(2)			
RT^c^				9.6(8)		2.682(7)	0.013(1)			

RT^c^ is the room temperature after being heated up to 500  °C and cooled down.

Data are shown in Fig. 4a. *N* is the coordination number, *d* is the distance, and σ^2^ is the Debye–Waller factor of atomic pairs. *S*_0_^2^ = 0.86 was determined by fitting the EXAFS data of a Pt foil and used in the other fittings.

**Table 2:** Fit results of EXAFS data of H_2_O_2_-Pt/Ti–FS at the Pt L_3_ edge.

T ( °C)	Pt–O			Pt-Pt(1)				Pt-Pt(2)		
	*N*	*d* (Å)	σ^2^ (Å^2^)	*N*		*d* (Å)	σ^2^ (Å^2^)	*N*	*d* (Å)	σ^2^ (Å^2^)
RT	5.7(7)	2.003(7)	0.003(1)					8(1)	3.28(1)	0.018(2)
100	5.7(7)	2.010(7)	0.004(1)					8(1)	3.22(4)	0.026(5)
150	3.8(7)	2.016(7)	0.004(1)	2(1)		2.75(1)	0.014(1)	6(1)	3.05(1)	0.024(2)
200	1(1)	2.05(1)	0.006(1)	8(1)		2.719(7)	0.014(1)			
250				7.9(8)		2.713(6)	0.012(1)			
300				7.6(8)		2.702(7)	0.012(1)			
350				7.2(8)		2.699(7)	0.011(1)			
400				6.8(8)		2.702(8)	0.011(1)			
450				7.2(8)		2.708(8)	0.013(1)			
500				7.8(9)		2.702(7)	0.014(1)			
RT^c^				7.6(9)		2.730(8)	0.008(1)			

Data are shown in Fig. 4b.

The dispersion and the structural stability of Pt nanoparticles can be affected by supports. TEM images show that lump Pt particles form on FS supports, while small and uniform Pt nanoparticles are spread over at Ti–FS supports, as shown in Fig. 2e–g. This agrees well with previous observations^9,10^. Previous EXAFS measurements revealed that most of the Pt nanoparticles bond with the Ti atoms of the Ti–FS supports^11^. This indicates that most of the Pt atoms choose TiO_x_ complexes rather than FS as supports, when the uniform mixture of the Pt precursor and the calcined Ti–FS powder is heated up to 500  °C. It is noted that the weight ratio of the Ti precursor and the FS was 2:1. The EXAFS data of Ti–FS and Pt/Ti–FS with and without H_2_O_2_ treatment at the Ti K edge were also analyzed in the same manner as the EXAFS data analysis at the Pt L_3_ edge, and the best fit results are summarized in Table 3. The best fit of EXAFS data at the Ti K edge suggests that the Ti atoms of Ti–FS form Ti–O complexes in the temperature range of RT – 500  °C. When an H_2_O_2_ treatment is applied on the Ti–FS, the local structure around the Ti atoms is substantially changed, compared to that of the Ti–FS at RT^c^; however, TiO_x_ do not form into a crystalline structure, as shown in Fig. 4c D. It is noted that the Ti–FS was calcined at 500  °C before the H_2_O_2_ treatment. The EXAFS data of Pt/Ti–FS and H_2_O_2_-Pt/Ti–FS at the Ti K edge shows the Ti–O(1), Ti–O(2), Ti–Ti(1), and Ti–Ti(2) pairs. This means that the Ti atoms of Pt/Ti–FS and H_2_O_2_-Pt/Ti–FS form a TiO_2_ crystalline structure, although there is still a substantial amount of structural distortion in the atomic pairs. TiO_x_ complexes with Ti^4+^ were also observed by H_2_O_2_ treatment^38,39^. In an anatase TiO_2_, a Ti atom consists of a Ti–O octahedron with six O atoms. The slightly low coordination of O atoms of Pt/Ti–FS and H_2_O_2_-Pt/Ti–FS indicates the existence of some vacancies on the O sites of the octahedrons. The coordination numbers of both the O and Ti atoms of the Pt/Ti–FS and H_2_O_2_-Pt/Ti–FS remain constant during heating. For the Pt/Ti–FS, the low coordination of Ti atoms and the large σ^2^ value of Ti–Ti pairs, particularly at 250  °C, indicate an incomplete TiO_2_. It is worth noting that σ^2^ values can be gradually increased at temperatures above 100 K due to the thermal vibration of atomic pairs^42,51^. Compared to that at RT, the σ^2^ value of the Ti–Ti pairs of H_2_O_2_-Pt/Ti–FS decreased when heated up to 250  °C. This finding strongly suggests that the TiO_2_ particles of H_2_O_2_-Pt/Ti–FS form into a more stable crystalline structure at 250  °C. The distance change of the Ti–O and Ti–Ti pairs of H_2_O_2_-Pt/Ti–FS serves as further evidence of TiO_2_ crystallization at 250  °C, compared to that at RT. The EXAFS measurements at the Ti K edge reveal that the Ti atoms form a distorted-anatase TiO_2_ in H_2_O_2_-Pt/Ti–FS, whereas the Ti atoms do not form a stable crystalline structure in Pt/Ti–FS. The in-situ EXAFS measurements indicate that the Pt precursor and the H_2_O_2_ treatment in succession assist the Ti atoms of Ti–FS in forming into a crystalline structure.

**Table 3:** Fit results of EXAFS data of anatase TiO_2_ powder, Ti–FS, H_2_O_2_–Ti–FS, Pt/Ti–FS, and H_2_O_2_-Pt/Ti–FS at the Ti K edge.

Specimen	T ( °C)	Ti–O(1)			Ti–O(2)			Ti–Ti(1)			Ti–Ti(2)		
		*N*	*d* (Å)	σ^2^ (Å^2^)	*N*	*d* (Å)	σ^2^ (Å^2^)	*N*	*d* (Å)	σ^2^ (Å^2^)	*N*	*d* (Å)	σ^2^ (Å^2^)
Anatase TiO_2_	RT	2.2(3)	1.859(5)	0.003(1)	4.3(5)	1.964(5)	0.003(1)	4.0(3)	3.044(5)	0.006(1)	4.0(3)	3.791(5)	0.006(1)
Ti–FS	RT	3.7(7)	1.946(8)	0.010(1)				2(1)	3.01(1)	0.013(1)			
	500	3.2(7)	1.884(8)	0.007(1)				2(1)	2.96(2)	0.019(4)			
	RT^c^	3.2(7)	1.903(8)	0.005(1)				2(1)	3.01(1)	0.014(2)			
H_2_O_2_–Ti–FS	RT	1.6(7)	2.17(1)	0.005(1)	3.2(7)	1.988(8)	0.005(1)	2.8(7)	3.33(1)	0.008(1)			
Pt/Ti–FS	RT	1.6(4)	1.816(5)	0.003(1)	3.2(8)	1.993(5)	0.003(1)	3(1)	3.00(1)	0.012(1)	3(1)	3.94(1)	0.009(1)
	250	1.6(4)	1.815(4)	0.003(1)	3.6(8)	1.994(7)	0.003(1)	3(1)	3.00(1)	0.010(1)	3(1)	3.76(4)	0.020(7)
H_2_O_2_-Pt/Ti–FS	RT	1.6(4)	1.973(5)	0.010(1)	3.2(8)	1.982(5)	0.010(1)	3(1)	2.99(2)	0.014(2)	4(1)	3.94(2)	0.017(4)
	250	1.6(4)	1.943(5)	0.012(1)	3.6(8)	1.952(8)	0.012(1)	3(1)	2.90(1)	0.010(1)	4(1)	3.71(1)	0.009(2)

Data are shown in Fig. 4c. *S*_0_^2^ = 0.83 was determined by fitting the EXAFS data of the anatase TiO_2_ powder and used in the other fittings.

## Discussion

### DFT calculations of Pt atoms on TiO_2_ surfaces

**Figure 6:**
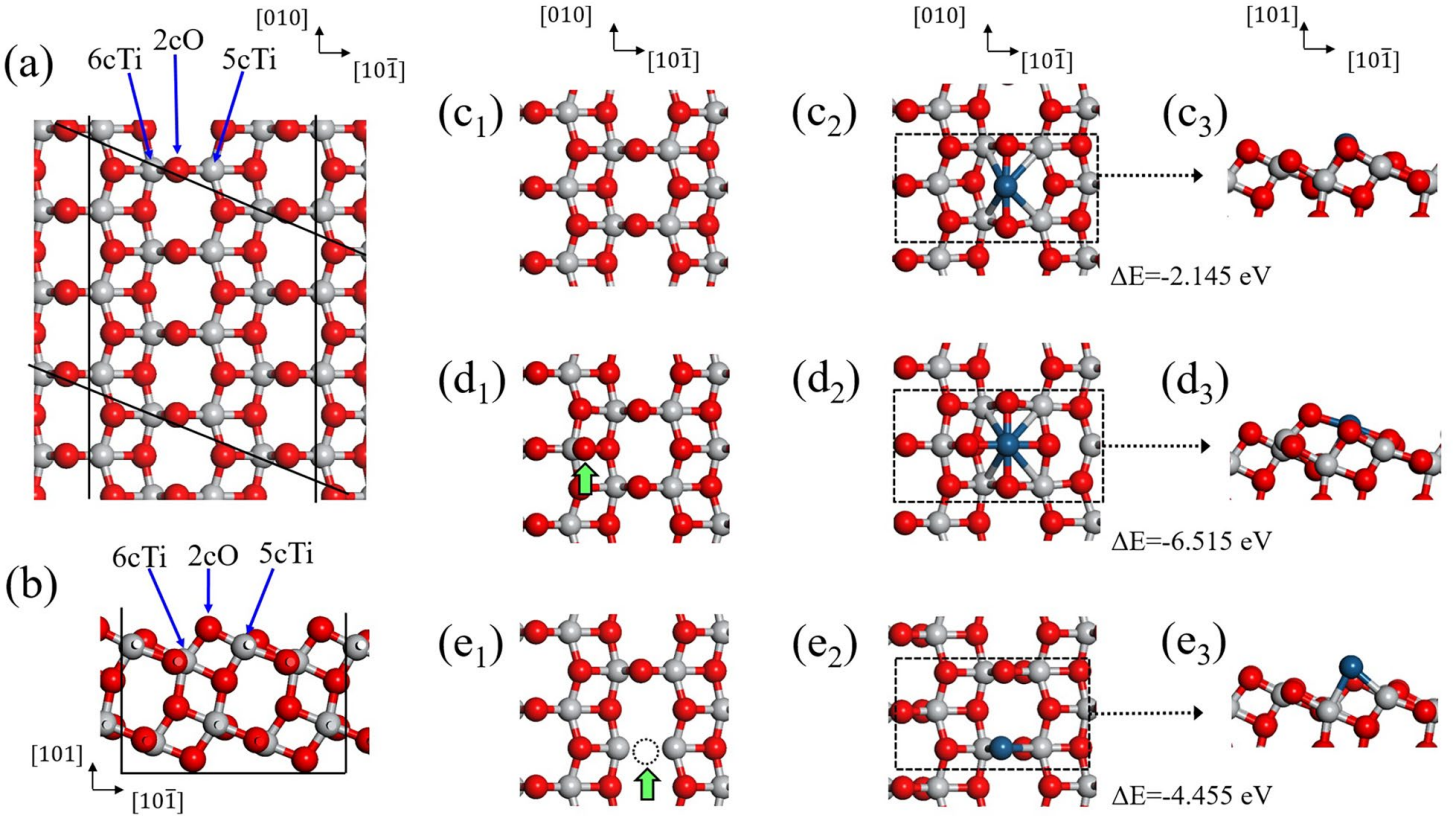
(**a**) and (**b**) Top and side views of anatase TiO_2_(101) surface, respectively. Top views of (**c**_**1**_) bare–TiO_2_(101), (**d**_**1**_) oxidized–TiO_2_(101), and (**e**_**1**_) reduced–TiO_2_(101) surfaces, respectively. The green vertical arrows in (**d**_**1**_) and (**e**_**1**_) indicate added (oxidized–TiO_2_) and removed (reduced–TiO_2_) O atoms, respectively. DFT calculation results with a Pt adatom on (**c**_**2**_) bare–TiO_2_(101), (**d**_**2**_) oxidized–TiO_2_(101), and (**e**_**2**_) reduced–TiO_2_(101) surfaces, respectively. ΔE is the total energy change due to a Pt adatom, and it is described in the text. (**c**_**3**_), (**d**_**3**_), and (**e**_**3**_) are the side views of (**c**_**2**_), (**d**_**2**_), and (**e**_**2**_), respectively. The gray, red, and blue symbols are Ti, O, and Pt atoms, respectively.

The interfacial structures of Pt atoms and TiO_2_ supports certainly affect the dispersion and the stability of Pt nanoparticles. Based on the EXAFS measurements, DFT calculations were performed using the CASTEP code in the Materials Studio to understand the bonds between the Pt atoms and TiO_2_ supports at the interfaces^58^. For the DFT calculations, the TiO_2_(101) surface is chosen because its surface has better thermodynamic stability than the other surfaces^59–61^. A 2 × 3 supercell slab with twenty-four TiO_2_ molecular units, which includes four Ti layers and eight O layers, is used in this study to construct the anatase TiO_2_(101) surface that is similar to those used in previous studies^62–64^. It has dimensions of 11.4 Å × 11.4 Å × 20 Å containing a vacuum region of about 15 Å to avoid interactions between the periodically-repeated slabs and to separate the slabs. Figure 6a and b show a part of the TiO_2_(101) surface of the supercell slab. In the calculations, the atoms of the bottom two layers—i.e., the lower half of the slab—are fixed at the original positions of anatase TiO_2_, and the rest of the atoms are allowed to freely move their positions to minimize the total energy of the system. A Pt atom is added on three different TiO_2_(101) surfaces, such as bare–TiO_2_, oxidized–TiO_2_, and reduced–TiO_2_, as shown in Fig. 6c–e, respectively. Oxidized- and reduced–TiO_2_(101) surfaces are generated by adding one O atom on the top of the 5cTi atom and by removing the bridging O atom (2cO), respectively, as shown in Fig. 6d and e, respectively^65^. Pt adatoms are initially placed in the middle of the hexagons of the TiO_2_(101) surfaces in Fig. 6c1, d1, and e1. After DFT calculations, the final locations of the Pt atoms are shown in Fig. 6c2, c3, d2, d3, and e2, e3.

Before the DFT calculations, we first examined the cutoff energy of plane waves in the range of 300–700 eV and the k-point grid using the Monkhorst–Pack method to reduce the computation time and obtain sufficient precision. The ultra-soft pseudopotentials with a cutoff energy of 550 eV, 3 × 3 × 2 k-point grids, and Perdew-Burke-Ernzerhof (PBE) functional of the generalized gradient approximation (GGA) are used^66^. The top layers of the slab including Pt adatoms and overlayers are relaxed until the force is less than 0.1 eVÅ^-1^ in the Cartesian coordinates. The calculation is terminated when the total energy difference between the present and final calculations converges to less than 5 × 10^–5^ eV per atom. The binding energy of a Pt atom on TiO_2_ is defined as △E = E_Pt/TiO2_ − E_TiO2_ − E_Pt_, where E_Pt/TiO2_ is the total energy of a Pt atom on TiO_2_; E_TiO2_ is the total energy of a TiO_2_ slab for bare–TiO_2_, oxidized–TiO_2_, and reduced–TiO_2_ cases; and E_Pt_ is the total energy of a single Pt atom. The DFT calculations show △E =  − 2.145 eV, − 6.515 eV, and − 4.455 eV for bare TiO_2_, oxidized TiO_2_, and reduced TiO_2_, respectively. This result indicates that Pt atoms more strongly bond to oxidized or reduced TiO_2_ than they do to bare TiO_2_. A strong bond of Pt atoms to reduced–TiO_2_ surface corresponds to a strong metal-support interaction (SMSI). Previous studies have shown that the catalysis efficiency of noble-metal on transition-metal-oxide supports decreases by reduction at a high temperature due to an SMSI^67–70^. The DFT calculations show that the bond of Pt atoms to oxidized-TiO_2_ is more stable than the bond of Pt atoms to the others, as shown in Fig. 6. In this study, we first report a strong bond of Pt atoms to oxidized–TiO_2_ surfaces, which is the origin of the high dispersion and high stability of Pt atoms on Ti–FS supports, based on the EXAFS measurements and the DFT calculations.

### Wavelet-transformed EXAFS analysis

**Figure 7:**
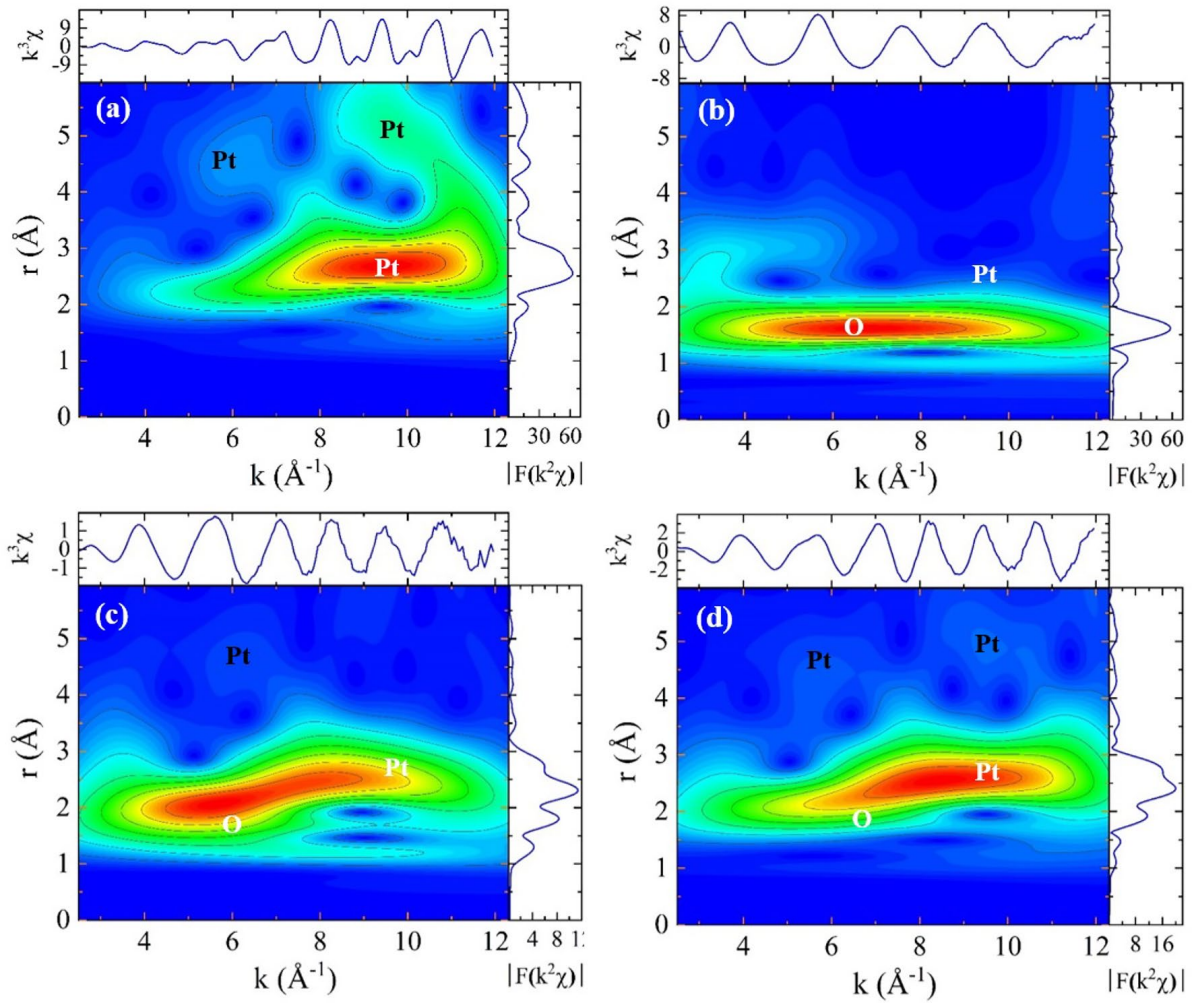
At Pt L_3_ edge, WT-EXAFS of (**a**) Pt foil at RT, (**b**) RT, (**c**) 200  °C, and (d) RT^c^ of H_2_O_2_-Pt/Ti–FS. The top and the right data are the measured EXAFS in the k-space and FT-EXAFS in the r-space, respectively.

The DFT calculations suggest that there are Pt-O bonds at the interfaces of Pt/TiO_2_ during the processes of the H_2_O_2_ treatment and the heating above 250  °C. Since Fourier-transformed (FT) EXAFS analysis does not show a distinguishable feature of Pt-O pairs, as shown in Fig. 5, we performed the wavelet-transformed (WT) EXAFS analysis which has structural information in the k-space as well as in the r-space. Figure 7 shows the WT-EXAFS images of the Pt foil and the H_2_O_2_-Pt/Ti–FS at different temperatures. The EXAFS signal of the Pt atoms of the Pt foil in the k- and r-spaces is quite different from that of the O atoms around a probing Pt atom of the H_2_O_2_-Pt/Ti–FS, as shown in Fig. 7a and b, respectively. The signals of WT-EXAFS near 1.5 Å and 2.5 Å correspond to the nearest O and Pt atoms around the probing a Pt atom, respectively. The WT-EXAFS signals of the O and Pt atoms are horizontally distributed around the peaks at ~ 6.5 Å^-1^ and ~ 9.5 Å^-1^, respectively. Figure 7b shows that the O signal of the H_2_O_2_-Pt/Ti–FS is dominant over the Pt signal at the RT. When the H_2_O_2_-Pt/Ti–FS is heated up to 200  °C, the O signal decreases and the Pt signal clearly appears. This agrees well with the FT-EXAFS results, as shown in Table 2. Figure 7d shows a weak but definite signal of O atoms. This indicates the presence of Pt-O pairs of H_2_O_2_-Pt/Ti–FS at RT^c^. After the reduction process of heating up to 500  °C in an H_2_ environment, the absence of oxygen atoms is expected at the surfaces of Pt nanoparticles. The WT-EXAFS signal of Pt-O pairs at RT^c^ likely indicates Pt-O bonds at the interface of Pt/TiO_2_. This corresponds to the DFT calculations. Since the EXAFS signal of Pt-O pair of H_2_O_2_-Pt/Ti–FS at RT^c^ is considerably weak, it may not be fully detected by the Fourier transformed EXAFS due to the resolution limit.

The mean diameters of the Pt nanoparticles of H_2_O_2_-Pt/Ti–FS and Pt/Ti–FS are estimated to be ~ 11 Å and ~ 22 Å for the Pt coordination numbers of 7.9 and 9.6, respectively, using a hemispherical model^11^. The mean diameters suggest that an H_2_O_2_ treatment prevents the agglomeration of Pt atoms on TiO_2_ at high temperatures. The SMSI effects of noble metal/transition-metal oxides have been observed on several different systems^67–70^. Heterogeneous catalysts of noble-metal nanoparticles/transition-metal-oxide supports are widely used for practical applications^24–27^. With an H_2_O_2_ treatment, oxygen atoms penetrate into Pt/Ti–FS and form the bond of Pt-O–Ti at the interface of Pt/TiO_2_, as shown in Fig. 6d2 and d3. The interfaces of Pt atoms and TiO_2_ supports are initially somewhat unstable due to dangling bonds of the TiO_2_ surface. When H_2_O_2_ is applied to Pt/TiO_2_, an additional oxygen atom forms a stable Pt-O–Ti bond at the interface, thus reducing the surface energy. The additional oxygen atoms at the interface simultaneously bond with Pt and Ti atoms, and they form a Ti–O octahedron. As a result, the Pt atoms on Ti–FS supports are restricted and do not aggregate at high temperatures. A lack of change of the Pt coordination numbers of H_2_O_2_-Pt/Ti–FS in the temperature range of 200–500  °C and at RT^c^, as shown in Table 2, indicates that the bonds of Pt atoms to TiO_2_ surfaces are considerably strong and remain constant even at 500  °C. This finding is consistent with the conversion results of the heterogeneous catalysts of H_2_O_2_-Pt/Ti–FS^9,71^.

Oxidation due to H_2_O_2_ treatments has been observed in many different transition-metal systems, including Co, Zr, Mn, Ti, Zn, and Cr^30–33,35,36,40^. Imamura and co-workers suggested that H_2_O_2_ molecules on a metal surface are converted into hydroxyl anions and hydroxyl radicals (^*^OH) as follows^72^, H_2_O_2_ + e^-^ → *OH + [OH]^-^. Previous studies have shown that an H_2_O_2_ treatment changes the surface morphology of metals, assists the bonds of heterogeneous metal atoms, and causes oxidation of metal surfaces^30–33,35,36,40^. Our XAFS measurements and DFT calculations are consistent with the previous reports. When the Pt precursors are embedded into Ti–FS supports during the synthesis of Pt/Ti–FS catalysts, they randomly bond to the surfaces of TiO_x_ complexes. Our XAFS measurements indicate that the Pt precursor induces the TiO_x_ of the Ti–FS supports to an unstable-distorted TiO_2_ and that a subsequent H_2_O_2_ treatment assists it to be a stable-distorted TiO_2_. The H_2_O_2_ treatment to the Pt/Ti–FS supplies additional oxygen atoms to both Pt and Ti atoms. The additional oxygen atoms prevent the aggregation of Pt atoms and bond with Pt and Ti atoms at the interface of Pt/TiO_2_. When H_2_O_2_-Pt/Ti–FS is heated in an H_2_ environment, the O atoms gradually dissociate from the Pt atoms, and the deoxidized Pt atoms form into structurally-stable Pt nanoparticles with a uniform size on the rough TiO_2_ supports, which becomes a more stable structure at high temperatures. The additional O atoms stemming from the H_2_O_2_ treatment tightly grasp Pt atoms on TiO_2_ supports. This is consistent with the EXAFS measurements that show a lack of change of local structures of Pt nanoparticles at RT^c^, compared to those at 500  °C. Our results of the Pt/TiO_2_ interface correspond to inactive and structurally stable transition-metal oxides with the extra O atoms which exist at the outermost boundaries^73–75^. When the process of the Pt impregnation and the H_2_O_2_ treatment is turned out of order; the H_2_O_2_ treatment is first applied on calcined Ti–FS supports and then, subsequently, the Pt precursor is embedded on H_2_O_2_/Ti–FS, the dispersion and structural stability of Pt-H_2_O_2_/TiO_2_ (data not shown here) were nearly the same as the results of H_2_O_2_-Pt/Ti–FS. This indicates that the dispersion and structural stability of Pt/TiO_2_ are independent of the order of an H_2_O_2_ treatment before and after the Pt precursor are embedded on Ti–FS supports. Previous studies showed that the Pt catalyst performance of H_2_O_2_-Pt/Ti–FS was considerably enhanced, compared to that of Pt/Ti–FS^9^ and that the stability of reaction performance of Pt/Zr-Si was also enhanced due to H_2_O_2_ treatment^76^. Our study and the previous observation evidence that H_2_O_2_ treatment enhances the dispersion and the structural stability as well as the reaction performance and stability of Pt/transition-metal supports.

## Conclusions

XAFS measurements and DFT calculations show that the additional oxygen atoms at the interface of Pt/TiO_2_ play an important role in the dispersion and structural stability of Pt nanoparticles. The additional oxygen atoms can be simply supplied by an H_2_O_2_ treatment. XAFS measurements at the Pt L_3_ and Ti K edges reveal that, when H_2_O_2_-Pt/Ti–FS is heated above 250  °C in an H_2_ environment, Pt atoms form into stable nanoparticles with a high dispersion on TiO_2_ supports. The heterogeneous catalysts of Pt/TiO_2_ with H_2_O_2_ treatment are structurally quite stable without Pt aggregation in the temperature of RT – 500  °C. DFT calculations suggest that a strong bond of Pt-O–Ti is formed at the interface of Pt/TiO_2_. WT-EXAFS confirms the presence of the Pt-O bonds which exist at the interface of Pt/TiO_2_. The results of this study show that H_2_O_2_ treatment is a new strategy for the synthesis of various heterogeneous catalysts of noble-metal/transition-metal-oxide systems with high dispersion. The structural stability issue of heterogeneous catalysts for practical applications can also be resolved using an H_2_O_2_ treatment.

## Methods

### Synthesis of Pt nanoparticles/Ti–FS supports

For in-situ XAFS measurements, fumed silica (FS) which was commercially obtained from Sigma-Aldrich was dehydrated with anhydrous ethanol with a purity of 99.9% and subsequently reacted with titanium butoxide (Sigma-Aldrich) with the purity of 99% dissolved in ethanol. Titania-incorporated fumed silica (Ti–FS) was obtained by washing with ethanol and subsequently drying at 80 °C. After Ti–FS was calcined at 500 °C for two hours, it was impregnated with tetraamineplatinum(II) nitrate ([Pt(NH_3_)_4_](NO_3_)_2_) with a purity of 99%, which was purchased from Sigma-Aldrich, using an incipient wetness method. The catalysts of Pt on Ti–FS supports (Pt/Ti–FS) were dried at 80 °C. The Ti–FS and the Pt–Ti–FS were dissolved in H_2_O_2_ with the weight ratio of 1: ~ 20, stirred with magnetic bars until the mixed liquors became gel, and then dried at 80  °C to obtain the H_2_O_2_–Ti–FS and H_2_O_2_-Pt/Ti–FS specimens. The weight ratio of FS, titanium butoxide, and Pt precursor was 1:2: ~ 0.04. The synthesis procedures of the catalysts of Pt/transition-metal-oxide supports have previously been described in detail elsewhere^9,10^. In-situ XAFS measurements were performed from the Ti–FS, H_2_O_2_–Ti–FS, Pt/Ti–FS, and H_2_O_2_-Pt/Ti–FS at the Pt L_3_ and the Ti K edges during heating up to 500  °C because practical Pt/Ti–FS catalysts are obtained by calcining at 500  °C after the Pt precursor is impregnated to the calcined Ti–FS. The synthesis procedure of the specimens and the conditions of XAFS measurements are summarized in Fig. 1.

### EDS and TEM measurements

EDS measurements were performed to analyze the distribution of selected species atoms of the H_2_O_2_-Pt/Ti–FS specimen, as shown in Fig. 2. The EDS images show that Pt atoms are uniformly distributed over the entire sample. TEM images indicate that the size of Pt particles on FS is approximately 10 nm, meanwhile it is ~ 2 nm for the Pt/Ti–FS and H_2_O_2_-Pt/Ti–FS specimens. The size of Pt nanoparticles becomes more uniform when H_2_O_2_ is treated on Pt/Ti–FS. This agrees well with previous reports^9,10^.

### In-situ XAFS measurements

In-situ XAFS measurements were taken of Pt/Ti–FS specimens with and without H_2_O_2_ treatment at the Pt L_3_ edge (11,564 eV) and the Ti K edge (4,965 eV) with a transmission mode under H_2_ environment in the temperature range of room temperature (RT) – 500 °C. The XAFS measurements were carried out by selecting the incident X-ray energy with a three-quarters-tuned Si(111) double crystal monochromator at the 9BM beamline of the Advanced Photon Source (APS) and at the 8C beamline of the Pohang Light Source II (PLS II). To avoid self-absorption effects, the specimen powders were ground and sieved with a sieve having a mean size of 25 μm. The powders were homogeneously mixed with a boron-nitrite powder and pressed into a disk shape with a proper thickness in a hole of a copper sample holder for the absorption edge step sizes of 0.3–0.8 at both the Pt L_3_ and Ti K edges^37^. The specimens were maintained at a constant and uniform temperature during the XAFS scans.

## Data Availability

The datasets used and/or analyzed during the current study are available from the corresponding author on reasonable request.
